# Emerging roles of T helper 17 and regulatory T cells in lung cancer progression and metastasis

**DOI:** 10.1186/s12943-016-0551-1

**Published:** 2016-10-27

**Authors:** Erin A. Marshall, Kevin W. Ng, Sonia H. Y. Kung, Emma M. Conway, Victor D. Martinez, Elizabeth C. Halvorsen, David A. Rowbotham, Emily A. Vucic, Adam W. Plumb, Daiana D. Becker-Santos, Katey S. S. Enfield, Jennifer Y. Kennett, Kevin L. Bennewith, William W. Lockwood, Stephen Lam, John C. English, Ninan Abraham, Wan L. Lam

**Affiliations:** 1 Department of Integrative Oncology, British Columbia Cancer Agency, Vancouver, Canada; 2 Department of Pathology and Laboratory Medicine, University of British Columbia, Vancouver, Canada; 3 Departments of Microbiology and Immunology, University of British Columbia, Vancouver, Canada; 4 Department of Zoology, University of British Columbia, Vancouver, Canada; 5 British Columbia Cancer Research Centre Centre, Vancouver, Canada

**Keywords:** Th17, IL-17, Regulatory T cell, Treg, Lung cancer, Inflammation, Cancer immunology, Tumor microenvironment, Tumorigenesis, Prognosis

## Abstract

Lung cancer is a leading cause of cancer-related deaths worldwide. Lung cancer risk factors, including smoking and exposure to environmental carcinogens, have been linked to chronic inflammation. An integral feature of inflammation is the activation, expansion and infiltration of diverse immune cell types, including CD4^+^ T cells. Within this T cell subset are immunosuppressive regulatory T (Treg) cells and pro-inflammatory T helper 17 (Th17) cells that act in a fine balance to regulate appropriate adaptive immune responses.

In the context of lung cancer, evidence suggests that Tregs promote metastasis and metastatic tumor foci development. Additionally, Th17 cells have been shown to be an integral component of the inflammatory milieu in the tumor microenvironment, and potentially involved in promoting distinct lung tumor phenotypes. Studies have shown that the composition of Tregs and Th17 cells are altered in the tumor microenvironment, and that these two CD4^+^ T cell subsets play active roles in promoting lung cancer progression and metastasis.

We review current knowledge on the influence of Treg and Th17 cells on lung cancer tumorigenesis, progression, metastasis and prognosis. Furthermore, we discuss the potential biological and clinical implications of the balance among Treg/Th17 cells in the context of the lung tumor microenvironment and highlight the potential prognostic function and relationship to metastasis in lung cancer.

## Background

### Lung cancer

**Fig. 1:**
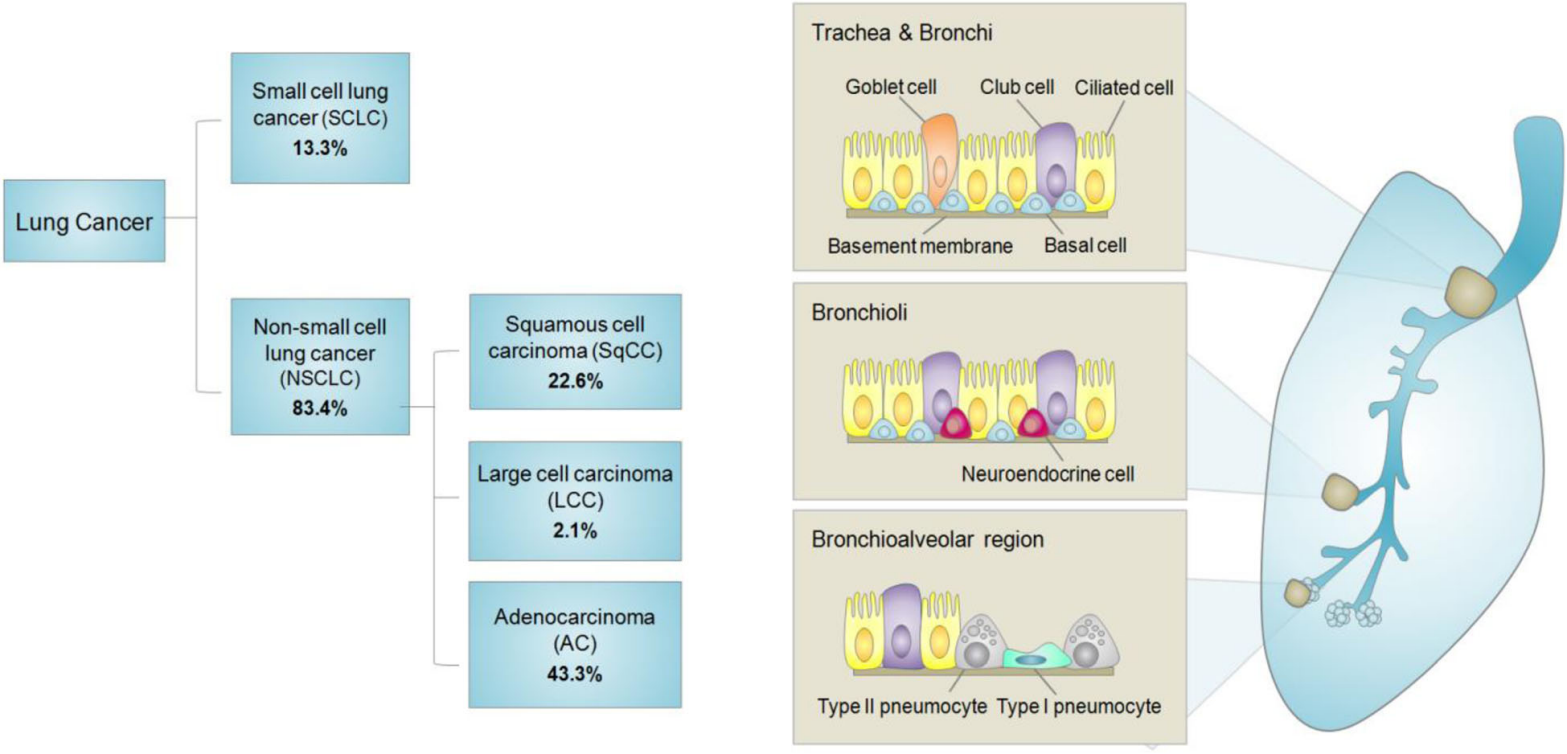
Percent incidence and typical histologies of lung cancer subtypes. Percent incidences shown are specific to American populations [5]. Locations of lung cancers depicted are generalized sites typical of lung tumorigenesis for subtypes. Lung cancers are classified into two major types: small cell lung cancer (SCLC) and non-small cell lung cancer (NSCLC). All forms of pulmonary carcinomas may be distributed throughout the lung but some locations are more typical for certain classes. SCLC primarily originates from central airways, and neuroendocrine cells are thought to be the precursors of this tumor type. As a heterogeneous disease, NSCLC is further subdivided into three major subtypes: squamous cell carcinoma (SqCC), large cell carcinoma (LCC) and adenocarcinoma (AC). Percentage distributions of NSCLC histologies total to NSCLC percentage (83.4 %), where remaining histogies (non-small cell carcinoma and other specified carcinomas) are not depicted. SqCC predominately originates from central airways and segmental bronchi and are thought to arise from basal cells. LCC are classified as tumors without general features associated with SCLC, SqCC and AC and may arise anywhere in the lung. The most common type of NSCLC is AC and is thought to principally arise from type II pneumocytes and club cells

Lung cancer is the leading cause of cancer-related deaths worldwide, with a dismal five-year survival rate of 17 % [1, 2]. There are two major types of lung cancer: small-cell lung cancer (SCLC), which accounts for ~15 % of lung cancer patients, and non-small-cell lung cancer (NSCLC), comprising the remaining ~85 % [3] (Fig. 1). The three major histological subtypes of NSCLC are adenocarcinoma (AC), squamous cell carcinoma (SqCC) and large cell carcinoma (LCC) (Fig. 1). AC is the most common histological subtype of lung cancer, accounting for approximately half of NSCLC cases (43.3 %; SEER Cancer Statistics Review, 1975–2012) and typically arises in the glandular epithelium of the lung periphery from either bronchioalveolar stem cells, club (formerly Clara) cells or type II pneumocytes [3–5] (Fig. 1). AC is also the predominant subtype that arises in patients who have never smoked [6]. By contrast, SqCC accounts for approximately 30 % of NSCLC (22.6 %; SEER Cancer Statistics Review, 1975–2012), develops primarily in the central airways and segmental bronchi, and strongly associates with a history of smoking [3, 5, 7] (Fig. 1). Regarding SCLC, the cell of origin has yet to be defined, but has been postulated to originate from differentiated neuroendocrine cell lineages, committed neuroendocrine progenitor cells or non-neuroendocrine cells that acquire neuroendocrine differentiation in the lung [8, 9] (Fig. 1).

### Inflammation and lung cancer

Inflammation has been shown to promote cancer initiation and progression [10]. Specifically, inflammatory programs have been implicated in all aspects of cancer development, including malignant transformation, cell proliferation and survival, angiogenesis, invasion and metastasis [11]. Studies have indicated a strong relationship between lung cancer risk factors and alterations in inflammatory cytokine levels, oxidative stress markers and immune cell composition.

#### Lung cancer risk factors, inflammatory cytokines and oxidative stress

Exposure to tobacco smoke is a principal risk factor associated with lung cancer — smokers display a 14-fold increased risk of developing lung cancer compared to never smokers [12]. Although lung cancer is often viewed as a smoker’s disease, if lung cancer in never smokers was considered as its own disease, it would rank as the seventh most common cause of cancer deaths worldwide and account for 300,000 deaths each year [6]. Other factors known to influence lung cancer risk include environmental carcinogens such as arsenic, radon, asbestos, air pollution, viral infection and genetic risk factors, including a family history of lung cancer [6, 13–16]. Furthermore, individuals with inflammatory lung disease, such as chronic obstructive pulmonary disease (COPD), have an elevated risk of developing lung cancer [17].

Cigarette smoking is known to drive altered local and systemic levels of inflammatory cytokines and reactive oxygen species (ROS) in the development of smoking related lung cancers. For example, inflammatory markers, including C-reactive protein (CRP), chemokine (C-C motif) ligand (CCL) 17, and CCL22, are elevated in the serum of former or current smokers compared to never smokers [18]. Furthermore, elevated levels of circulating inflammatory molecules CRP, CCL22, CCL17 and also chemokine (C-X-C motif) ligand (CXCL) 5, CXCL7, CXCL9, CXCL13 are associated with increased lung cancer risk in both current and former smokers [19]. Smokers with lung cancer have increased serum CCL20 levels, an inflammatory molecule shown to promote tumor cell proliferation and migration, that has also been significantly correlated with advanced disease and poor prognosis in lung cancer [20]. Surfactant protein D (SFTPD) is an important regulator of innate immunity, inflammation and oxidative stress secreted by type II pneumocytes in the airway; decreased expression of this protein in bronchoalveolar lavage (BAL) of smokers correlates with progression of bronchial dysplasia [21]. In addition to inflammatory cytokines and chemokines, ROS is also a major mediator of smoke induced damage, chronic inflammation and cancer development by promoting oxidative DNA damage and genomic instability [22]. Cigarette smoke is an enriched source of oxidants, which can enhance ROS generation by phagocytes to promote oxidative stress [23]. Increased recruitment of these phagocytes, including neutrophils and macrophages, is prominent in the lungs of smokers and patients with COPD compared to non-smokers [23]. Alternative oxidative stress markers, like extracellular superoxide dismutase (ECSOD), are elevated in the sputum of smokers [23, 24]. Taken together, alterations in cytokine and oxidative stress profiles of smokers indicate the presence of an important molecular link between smoking and lung inflammation.

In addition to smoking, exposure to airborne irritants and environmental carcinogens has been shown to induce lung inflammation. A well-documented example is asbestos, which refers to six unique silicate mineral fibers: chrysotile, amosite, crocidolite, tremolite, anthophyllite and actinolite. Inhaled asbestos fibers larger than 20 μm are not efficiently phagocytosed and remain in lung tissue, where they induce fibrosis, inflammation and eventually, carcinogenesis [15, 25]. Most of the inflammation driven effects of asbestos exposure are a consequence of increased ROS production [26]. The presence of asbestos fibers induces a chronic inflammatory response, which generates significant ROS that contributes to subsequent DNA damage [26, 27]. Interestingly, different classes of asbestos fibers can induce changes in cytokines detectable in the serum, including cytokines characteristic of a T helper 17 (Th17) immune response, which will be discussed further below. In mice, intratracheal exposure to chrysotile asbestos induces a pattern of chronic inflammation associated with Th1 cytokines, while amphibole asbestos exposure induces both a Th1 and Th17 cytokine response [28]. Additionally, it has been shown that tremolite and erionite (a fiber with similar characteristics to amphibole asbestos) exposure caused increased IL-17 in splenocyte cultures, and erionite exposure induced elevated serum IL-17 levels in mice [29].

Finally, respiratory diseases involving chronic inflammation are linked with lung cancer risk. Of note, patients with the inflammatory lung disease COPD have up to a tenfold increase of lung cancer risk, and COPD is linked to activation of pro-tumorigenic inflammatory signaling pathways in immune cells [30]. Tumor and immune cells can communicate through nuclear factor kappa-light-chain-enhancer of activated B cells (NF-κB) and signal transducer and activator of transcription 3 (STAT3)-dependent cytokine production. Oncogenic activation of STAT3 and NF-κB induces cytokine production by tumor cells that can regulate immunosuppressive and tumor-promoting functions of tumor infiltrating immune cells via trans-activation of these same transcription factors [31]. This molecular pathway is noted as a potential mechanism for COPD-related lung cancer development [31–33]. Taken together, these studies highlight that lung cancer risk is influenced by changes in systemic and local inflammation. Moreover, altered compositions and activities of immune cells modulate the tumor microenvironment to promote lung cancer development.

#### Inflammatory cells and lung cancer

**Fig. 2:**
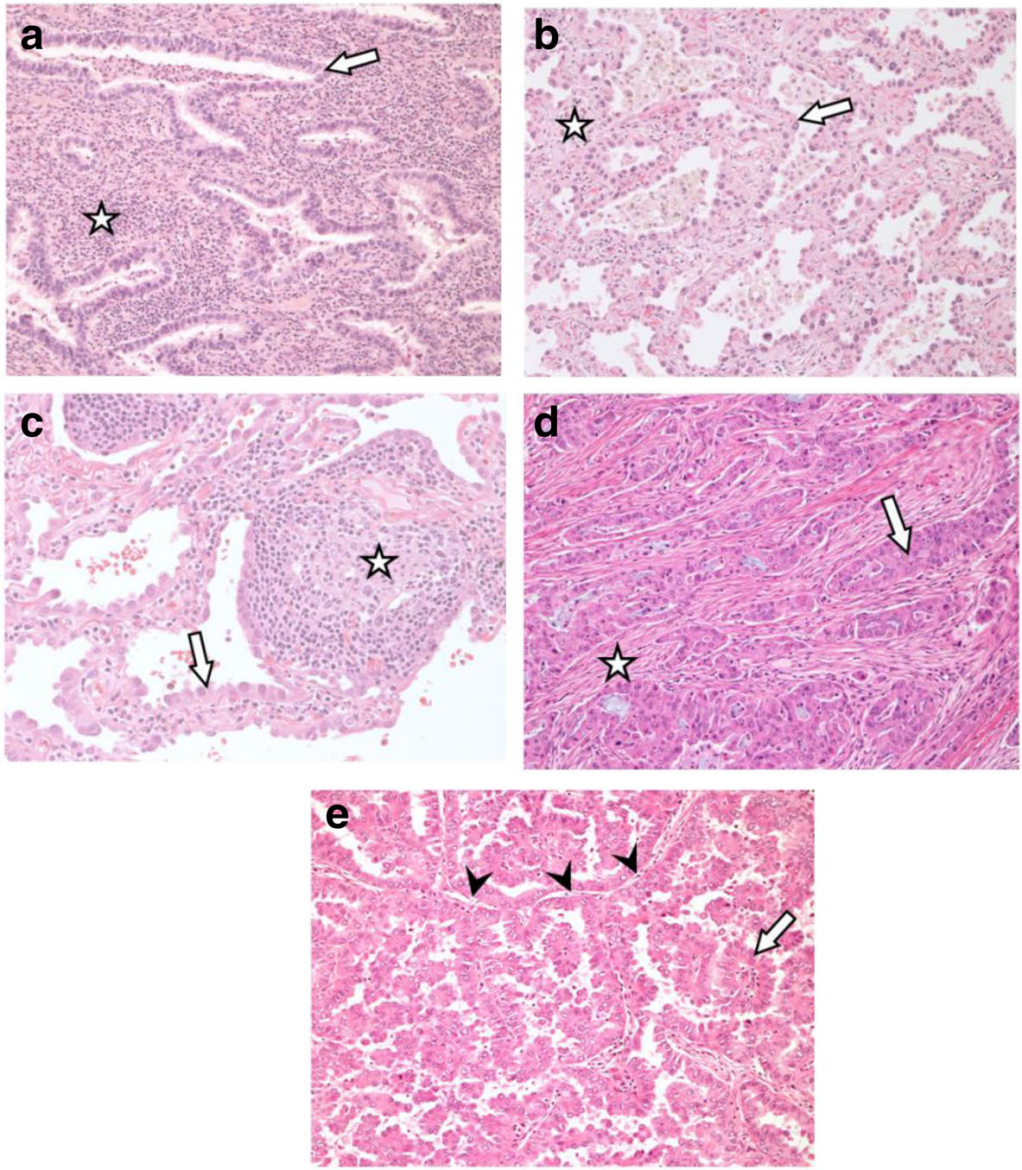
Variable immune cell infiltration within pulmonary AC in patients. **a** Lepidic growth pattern showing wide expansion (*star*) of alveolar interstitium by a diffuse population of mononuclear inflammatory cells, principally lymphocytes. Arrow indicates neoplastic cells. **b** A similar tumor showing minimal interstitial expansion (*star*) with few infiltrating inflammatory cells. Arrow indicates neoplastic cells. **c** Another lepidic growth region of AC showing focal expansion of the interstitium by lymphoid follicular hyperplasia (*star*). Arrow indicates neoplastic cells. **d** AC with infiltrating acinar pattern showing a desmoplastic (fibroblastic scarring) reaction (*star*) with very few infiltrating lymphocytes. Arrow indicates neoplastic cells. **e** AC with a papillary pattern demonstrating alveolar septae (*arrow heads*) with no fibrous expansion and no infiltration by lymphocytes. Arrow indicates neoplastic cells. Original magnification for images 100x

The biological process of inflammation relies on recruitment of diverse immune cell types. The inflammatory response depends on innate and adaptive immune cell activities to maintain tissue homeostasis [34]. However, immune cell infiltration is observed in tumors and can also promote cancer development, progression and metastasis [35] (Fig. 2). Immune cell composition in the tumor microenvironment may contribute to immune evasion and cancer development [36]. Innate and adaptive immune cells, including macrophages, neutrophils, natural killer (NK) cells and B cells, have been implicated in both anti-tumor and pro-tumor activities [37–43]. Specifically, the anti-tumor and pro-tumor roles of T cells in cancer development are currently of great interest. Therapeutic strategies targeting this adaptive immune cell type have been a major focus of recent immunotherapy development and applications, including for lung cancer treatment [44, 45].

T cells play diverse roles in the immune response and are highly relevant to lung cancer biology. CD8^+^ cytotoxic T lymphocytes (CTLs) facilitate immunosurveillance by T cell receptor (TCR) recognition of antigens bound to major histocompatibility complex (MHC)-I on antigen presenting cells and when activated, produce interferon gamma (IFN-γ), perforin and granzyme B that contribute to tumor cell cytolysis [46]. In addition to CD8^+^ CTLs, various CD4^+^ T cells, including regulatory T (Treg) and T helper 17 (Th17) CD4^+^ T cell subsets have emerged as key players across a variety of diseases involving inflammation, including cancer. Both these CD4^+^ T cell subsets facilitate a pro-tumor environment through the promotion and maintenance of an immunosuppressive and pro-tumor inflammation environment that could favor tumorigenesis, cancer progression and metastasis. Treg and Th17 subsets are generally thought to play opposing roles in regulating immunity, where cell fate determineration arises from a balance of transcription factors governing CD4^+^ T cell differentiation and Treg and Th17 cell generation [47]. For instance, mouse and human studies show that expression of the transcription factor forkhead box P3 (FoxP3) represses Th17 transcription factors retinoic acid receptor-related orphan receptor gamma t (RORγt) and ROR alpha (RORα) to drive Treg differentiation [48, 49]. Moreover, Th17 differentiation can occur when pro-inflammatory cytokines, including IL-6, IL-21 and IL-23, inhibit FoxP3 expression and subsequent repression of RORγt [48, 50]. In addition to *de novo* generation of Tregs from FoxP3^−^ T cells, Tregs can also be generated under homeostatic or pathological conditions via proliferation of thymus-derived FoxP3^+^ cells [51, 52]. Additionally, a novel mechanism of Treg-dependent promotion of Th17 differentiation via IL-2 sequestration has been shown to promote IL-17-driven inflammation and tumorigenesis in colon cancer, highlighting the complex interplay between these two cell types in the context of cancer [53].

## Main text

### Tregs and lung cancer

**Fig. 3:**
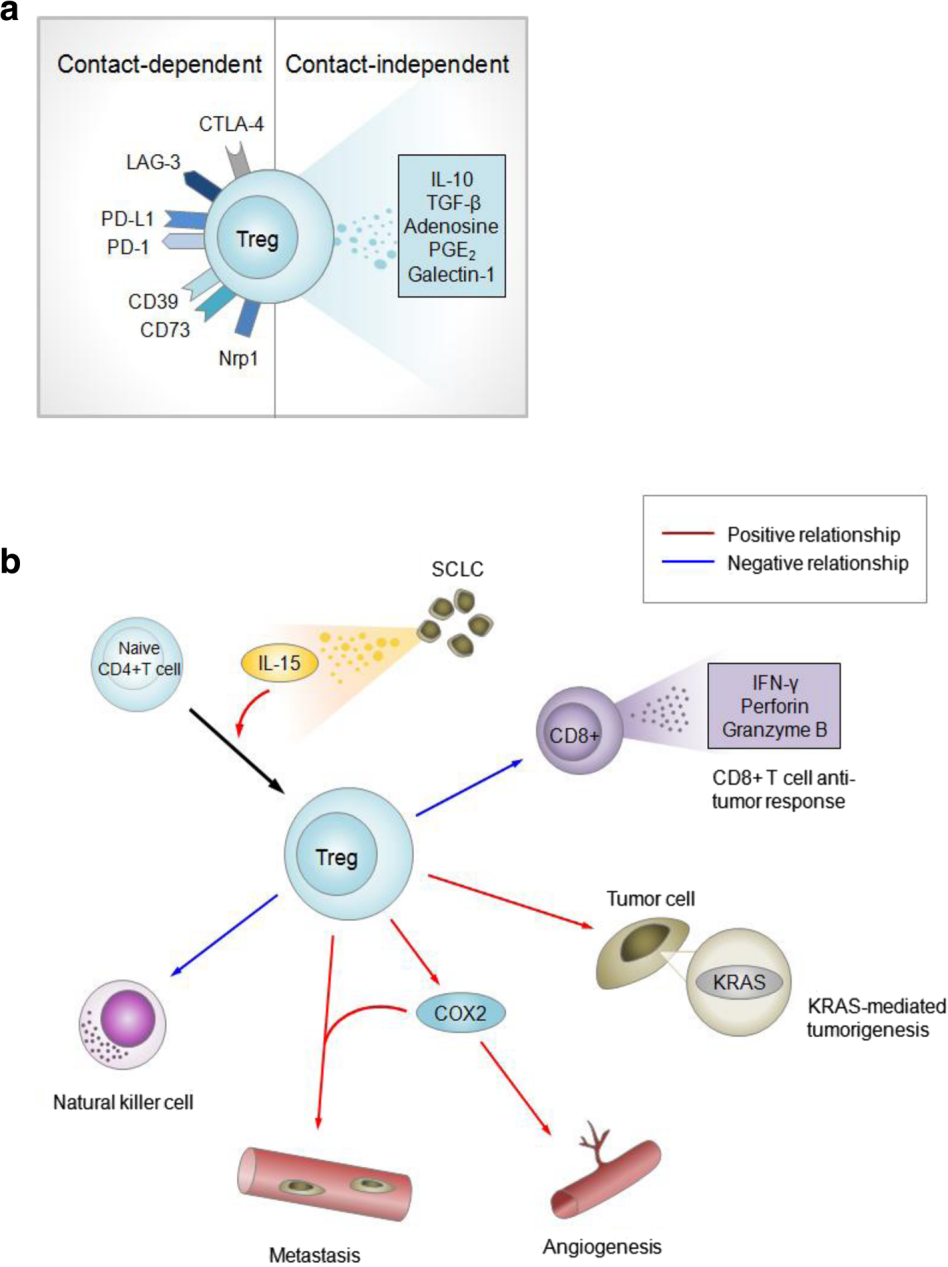
Potential roles of Tregs associated with lung cancer development. **a** Contact-dependent and contact-independent mechanisms of Tregs in mediating tumorigenesis. All receptors shown are mouse specific. For humans, receptors shown are human-specific except for LAG3, CD73 and Nrp1, which are non-human specific or where human specificity remains undetermined. **b** Immunosuppressive and pro-tumorigenic processes in lung cancer development depend on quantitative relationships of Treg populations. Arrows indicate Treg-dependent processes, with red indicating positive relationships and blue indicating negative Treg-dependent relationships

By maintaining tolerance toward innocuous antigens, Tregs represent a vital component of the adaptive immune system, which functions to prevent autoimmunity and chronic inflammation [54, 55]. Tregs represent a phenotypically diverse cell lineage classified according to their site of differentiation, either in the thymus or at extrathymic sites [56]. Although not definitive, these cells are generally characterized as CD4^+^CD25^high^, and express the master regulatory transcription factor FoxP3 [57]. Tregs can induce immunosuppression through contact-dependent mechanisms such as the expression of cytotoxic T-lymphocyte-associated protein 4 (CTLA-4), programmed cell death 1 (PD-1), programmed death-ligand 1 (PD-L1), lymphocyte-activation protein 3 (LAG-3), CD39/73 and neuropilin 1 (Nrp1), or through contact-independent mechanisms, including the sequestration of IL-2 and the production of the soluble immunosuppressive molecules IL-10, TGF-β, adenosine, prostaglandin E_2_ (PGE_2_) or galectin-1 [52, 55, 58–61] (Fig. 3a). In carcinogenesis, systemic expansion and intratumoral accumulation of immunosuppressive Tregs is thought to disrupt anti-tumor immunity, leading to the growth and metastasis of a variety of malignancies, including lung, breast, prostate and ovary [54, 56]. Certain cell surface molecules have been shown to have stabilizing effects on the Treg cell population: CD39 (ectonucleoside triphosphate disphosphohydrolase 1; ENTPD1) has been shown to increase stability of CD4^+^ FoxP3^+^ Tregs, contributing to their immunosuppressive function [62]. By suppressing anti-tumor effector cells, Tregs have emerged as active contributors to cancer progression [63, 64].

Tregs are implicated in the early stages of tumor development. In murine models of mutant Kras-driven AC, tumorigenesis was found to be Treg dependent, with Kras transgenic mice deficient in FoxP3^+^ Tregs developing 75 % fewer lung tumors [65] (Fig. 3b). Tobacco carcinogen exposure increased pulmonary FoxP3^+^ lymphocytes prior to tumor development, suggesting a potential role for Tregs in the generation of a favorable niche for the development of lung tumors driven by Kras, mutations mainly found in smoker-related lung cancers [65].

Tregs influence the tumor microenvironment during the progression of lung cancers. Murine models of lung AC have demonstrated that Tregs may inhibit CD8^+^ T cell-mediated anti-tumor immunity (Fig. 3b), with the depletion of Tregs resulting in tumor cell death and elevated levels of granzyme A, granzyme B, perforin and IFN-γ in infiltrating CD8^+^ T cells at early stages of tumorigenesis [66]. Further, the development of SCLC influences immunosuppressive activities of Tregs, where SCLC cell lines were reported to induce Treg generation from CD4^+^ T cells through the production of IL-15 [67] (Fig. 3b). In lung tumors, Tregs are also associated with expression of angiogenic and metastatic potentiator cyclooxygenase-2 (COX2), where elevated numbers of intratumoral FoxP3^+^ lymphocytes were positively correlated with high intratumoral expression of COX2, and can be induced by the tobacco carcinogen nicotine-derived nitrosamine ketone (NNK) in mouse lungs [68, 69] (Fig. 3b).

Emerging evidence suggests that Tregs promote metastasis and metastatic tumor foci development [52]. A clinical study of NSCLC observed that Treg levels in peripheral blood increased with stage and were highest in patients with metastatic tumors [70]. It was also reported that Treg levels were elevated in metastatic lymph nodes compared to nonmetastatic lymph nodes in patients with AC [71, 72]. Mouse models of Lewis lung carcinoma reveal that Tregs inhibit NK cell-mediated cytotoxicity in a TGF-β-dependent manner, and that depletion of Tregs contributes to enhanced NK cell antimetastatic activities [73] (Fig. 3b). Prognostically, the relative accumulation of Tregs in NSCLC tumors, and peripheral blood of SCLC patients (in relation to effector T cell populations) has been linked to increased risk of recurrence, and a high proportion of FoxP3^+^ lymphocytes in SCLC lung tumor biopsies correlates with poor survival [67, 68, 74, 75]. Another study in NSCLC identified elevated levels of intratumoral FoxP3^+^ lymphocytes were associated with reduced recurrence-free survival [68].

Taken together, these findings underscore the relevance of Tregs in promoting lung cancer progression and metastasis. In a clinical setting, targeting the immune checkpoint molecules CTLA-4 and PD-1 have recently received much attention in a variety of cancer types including lung cancers [76–79]. Ligation of these receptors leads to inhibition of T cell activation, particularly that of effector CD8^+^ T cells. The anti-CTLA-4 immunotherapeutic agent ipilimumab has demonstrated promising results in improving SCLC and NSCLC patient outcomes, while nivolumab (anti-PD-1 therapy) has been approved by the Food and Drug Administration (FDA) for the treatment of advanced squamous and non-squamous NSCLC [76–80]. However, the response rate to nivolumab was only 20 % in lung SqCC patients, and determining clinical biomarkers for treatment stratification for these available immunotherapies is a major focus [78, 81]. Currently, clinical biomarkers are limited for these treatments, as patients with little to no expression of these molecules in their lung tumors can still have beneficial therapeutic responses. For instance, patients harboring tumors with negative expression of the PD-1 ligand, PD-L1, can also benefit from anti-PD1/PD-L1 therapies, suggesting that clinical use of PD-L1 positivity as selection criteria could exclude patients who could potentially benefit from these treatments [61]. Other factors are at play and the molecular mechanisms underlying Treg recruitment and their immunosuppressive functions in the lung tumor microenvironment require further study to improve patient therapy and outcomes.

### Th17 cells and lung cancer

**Fig. 4:**
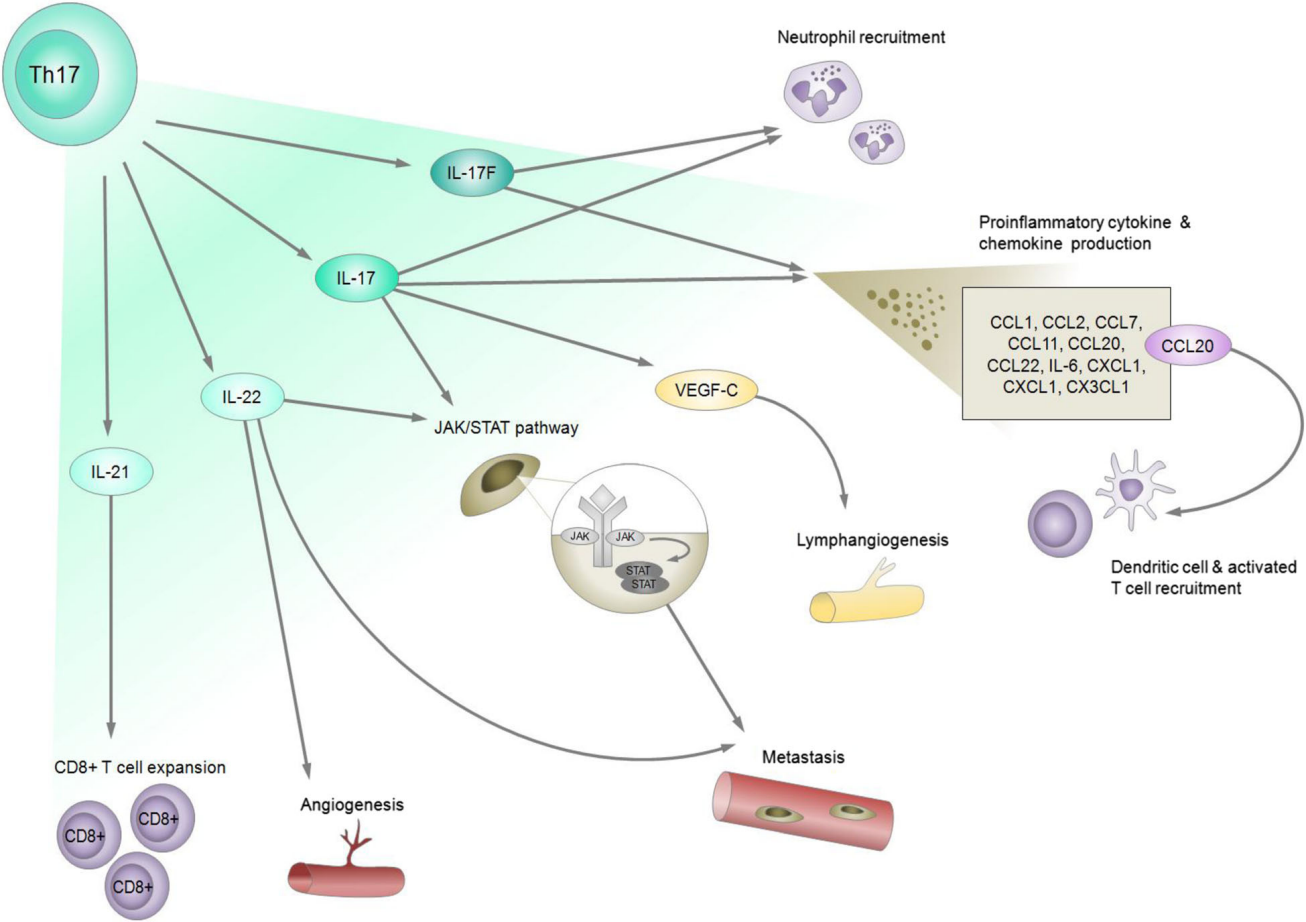
Pro-inflammatory and pro-tumorigenic roles of Th17 cells in lung cancer pathogenesis depend on Th17 cell cytokine production. Th17 cells are cardinal producers of IL-17, a family of pro-inflammatory cytokines orchestrating a variety of molecular mechanisms that promote lung tumorigenesis. For specific pro-inflammatory cytokine and chemokine expression stimulated by IL-17A or -F, refer to text in Section “Th17 cells and lung cancer”

Th17 cells are a group of CD4^+^ T helper cells that are phenotypically distinct from Th1 and Th2 cells, and have been characterized in many inflammatory lung diseases, including COPD [82–89]. Th17 cells express the transcription factors RORγt/RORC2 (mouse/human) and RORα, which drive Th17 differentiation and produce pro-inflammatory cytokines, including IL-17A, that modulate the tumor microenvironment [90, 91] (Fig. 4). The IL-17 cytokine family contributes to inflammation, cytokine and chemokine production, neutrophil recruitment in the context of lung inflammation and infection, and lung antitumor immunity [92, 93] (Fig. 4). Alterations to IL-17 and its signaling pathways are relevant to lung cancer development with IL-17 polymorphisms and epigenetic changes to the IL-17 signaling pathway correlating with increased predisposition to lung cancers [89, 94]. Thus, Th17 cells are an integral component of the inflammatory milieu in the tumor microenvironment, and may be causally involved in promoting distinct lung tumor phenotypes.

The IL-17 family of cytokines is comprised of six members: IL-17A (also known as IL-17), IL-17B, IL-17C, IL-17D, IL-17E (also called IL-25) and IL-17 F [93, 95]. Th17 cells produce IL-17A and IL-17 F [93, 96]. Serum IL-17 levels strongly associate with lung cancer development. Serum IL-17 levels were found to be significantly higher in NSCLC compared to subjects without cancer, which could potentially offer an additional diagnostic marker for NSCLC [97]. IL-17 cytokines contribute to inflammation by facilitating pro-inflammatory cytokine and chemokine induction. Exogenous treatment of mouse embryonic fibroblasts (MEFs) with either IL-17A or IL-17 F induced greater expression of *IL-6, CXCL1, CCL2* and *CCL7*. Elevated expression of chemokines *CCL7, CCL22, CCL20, CCL11* and *chemokine (C-X3-C motif) ligand 1 (CX3CL1)* were found in the lungs of transgenic mice chronically overexpressing IL-17A. In addition to upregulating these chemokines, elevated *CCL1, CCL2* and *CXCL1* expression was also present in the mouse lung epithelial cell line MLE12 upon exogenous IL-17A treatment [98] (Fig. 4).

**Fig. 5:**
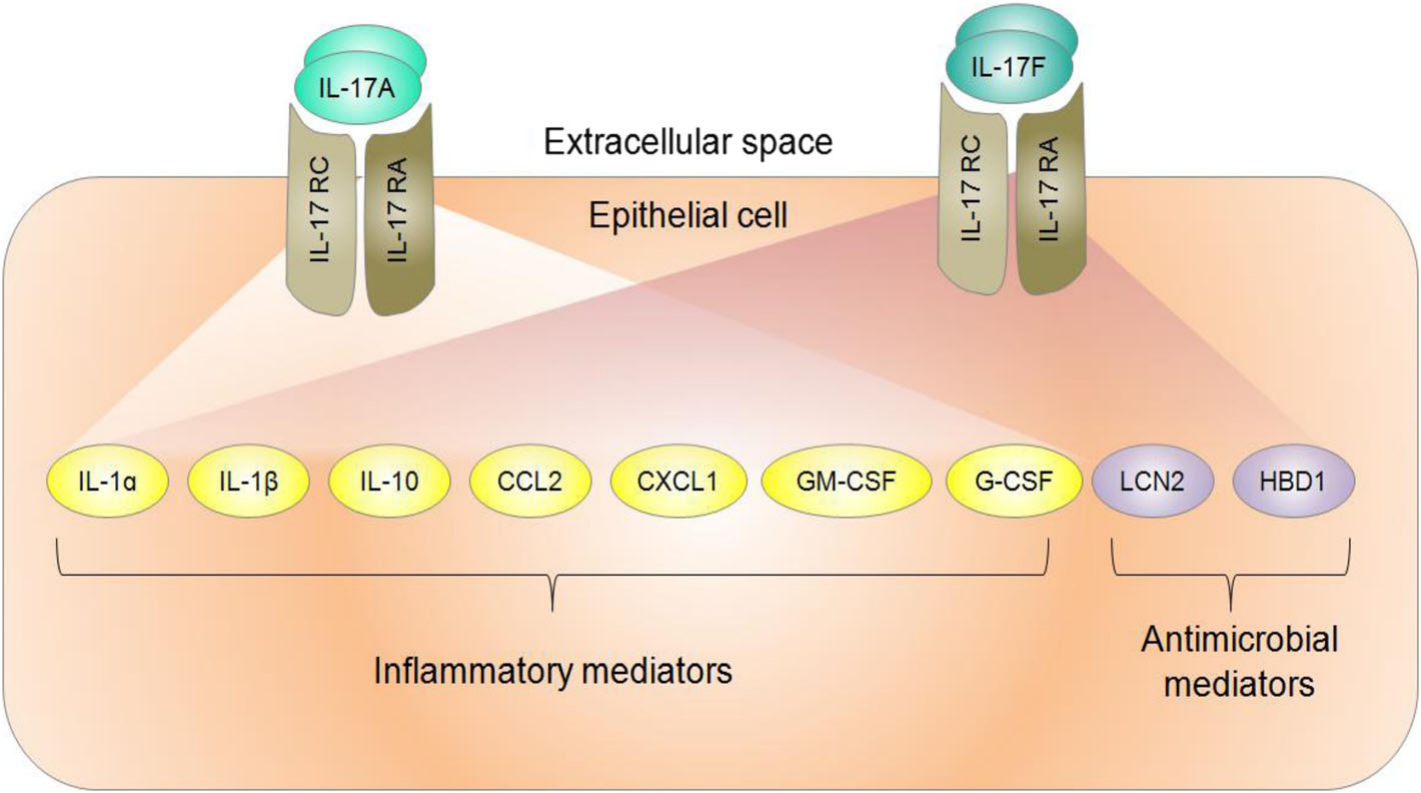
The IL-17 F signaling pathway is epigenetically altered in malignant COPD and non-malignant COPD lung airway epithelial cells. Top disrupted downstream molecular components of the IL-17 F pathway are involved in mediating inflammatory and anti-microbial processes. Genes involved in IL-17A signaling pathways and that overlap with deregulations in the IL-17 F signaling pathway are also depicted. CCL2: chemokine (C-C motif) ligand 2; CXCL1: chemokine (C-X-C motif) ligand 1; G-CSF: granulocyte colony-stimulating factor 3; CX3CL1: chemokine (C-X3-C motif) ligand 1, GM-CSF: granulocyte-macrophage colony-stimulating factor; HBD1: defensin beta 1; IL: interleukin; IL-17RA: interleukin 17 receptor A; IL- 17RC: interleukin 17 receptor C; LCN2: lipocalin 2

**Fig. 6:**
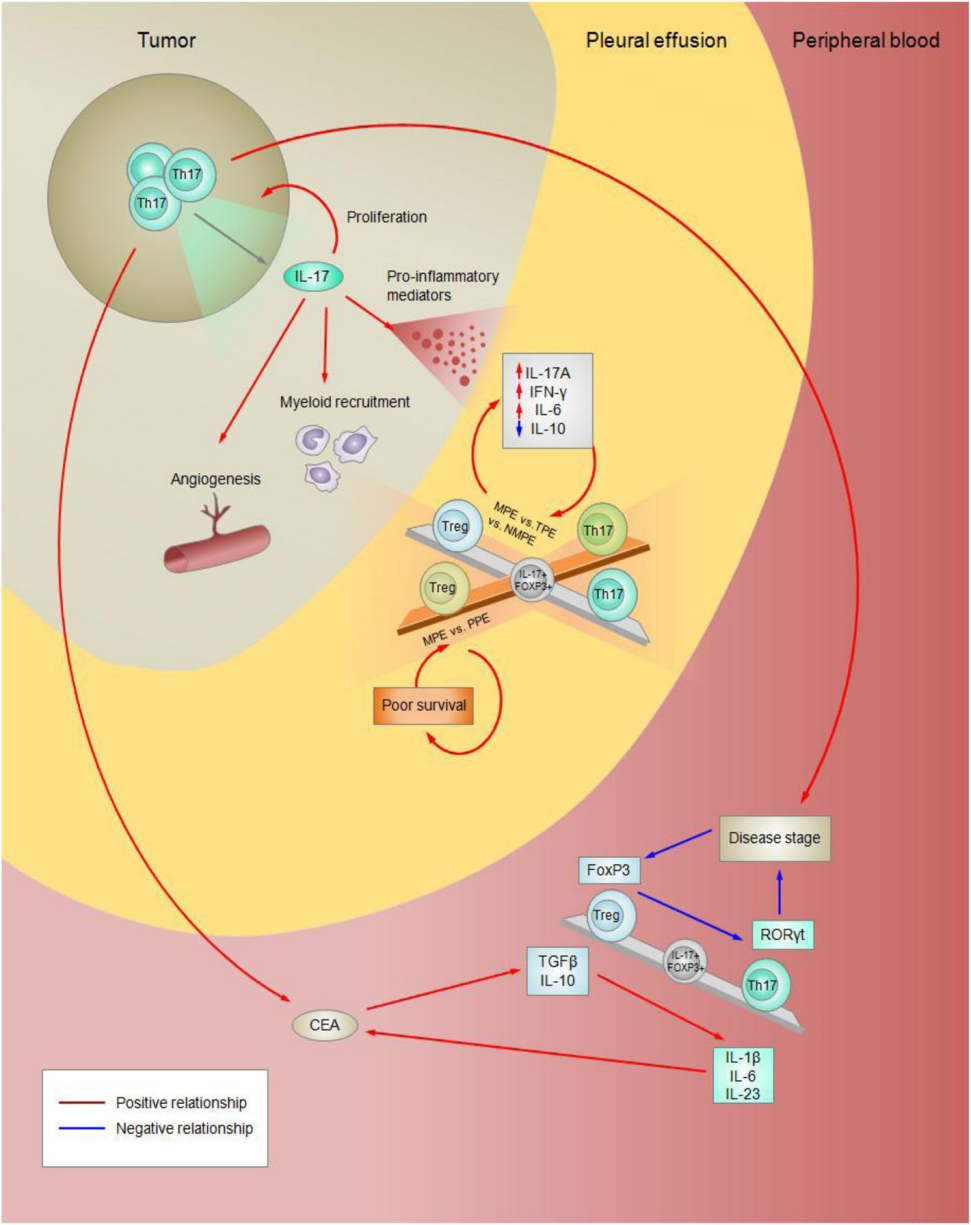
Treg/Th17 ratios are context-dependent in lung cancer patients and associate with disease pathogenesis and outcome of lung cancers. Blue arrows indicate negative relationships, and red arrows indicate positive relationships. Balance beams indicate correlation among Treg and Th17 cell subsets, with intermediate IL-17^+^FoxP3^+^ phenotypes present at the centre to indicate that these cells may contribute to cell ratios. The Treg/Th17 ratio has been primarily assessed in pleural effusion and peripheral blood in malignant and non-malignant pleural effusions. MPE: malignant pleural effusion; NMPE: non-malignant pleural effusion derived from non-chronic diseases; PPE: parapneumonic effusion; TPE: Tuberculous pleural effusion

Genetic variation and epigenetic alterations to the IL-17 F pathway may impact lung cancer development. Epigenetic analysis of DNA methylation patterns of COPD small airway epithelia has highlighted the relevance of the IL-17 F inflammatory response pathway in a disease significantly linked to lung cancer [89]. Specifically in COPD small airways, *IL-17 receptor C (IL17RC)* and *CXCL1* (upstream and downstream components of the IL-17 F inflammatory pathway, respectively) were both identified to be hypermethylated and underexpressed, while DNA hypomethylation and overexpression of *colony stimulating factor 2* (*CSF2),* an IL-17 F-induced pro-inflammatory cytokine, was observed [89]*.* Many other genes in the IL-17 F inflammatory pathway were also found to be altered by DNA methylation in COPD small airways, and alterations of these genes are known to contribute to carcinogenesis [99] (Fig. 5, Table 1). Single nucleotide polymorphisms (SNPs) in *IL-17 F* genes are significantly associated with lung cancer development. For example, the *IL-17 F* 7488G allele is associated with advanced stage or metastatic lung cancer in a Tunisian population [94]. COPD was characterized by increased Th17 cells (CD3^+^CD4^+^IL-17A^+^) in peripheral blood [100]. Likewise, high levels of Th17 cell cytokines have been observed in BAL of mouse models bearing oncogenic Kras-driven lung AC with concurrent induction of COPD-like inflammation through exposure to *Haemophilus influenzae* lysate (NTHi) [101]. *Il17* ^−/−^ mice had reduced lung tumor numbers, as well as reduced tumor cell proliferation, angiogenesis, myeloid cell recruitment and expression of pro-inflammatory mediators (*Il6*, *Cxcl2*, *Ccl2*, *Arg1*, *Csf3*, *Mmp7*, *Mmp12* and *Mmp13)* compared to *Il17* ^+/+^ mice (Fig. 6). Of note, reduction of lung tumor numbers occurred with IL-17 deficiency, but not with IL-17 F deficiency in lung tumor bearing mice [101]. Similarly, IL-17 has been shown to promote tumor growth in mice by increasing angiogenesis, metastasis and macrophage infiltration into tumors [102].

**Table 1:** Examples of genes in the IL-17 F pathway epigenetically altered in COPD and their respective roles in cancer

Epigenetically disrupted in COPD	Roles in cancer	References
IL-1α	Tumor cell-derived IL-1α increases tumor immunogenicity Precursor IL-1α from necrotic tumor cells promotes inflammation	[155]
IL-1β	Polymorphisms associated with overall cancer risk	[156]
IL-10	Polymorphisms associated with overall cancer risk Induces IFN-γ-mediated CD8^+^ anti-tumor immunity Treg cell-derived IL-10 suppresses Th17 inflammation	[157–159]
CCL2	Promotes metastasis and angiogenesis Recruits monocytes and macrophages contributing to inflammation	[160, 161]
CXCL1	Promotes metastasis, angiogenesis and cell proliferation Induces constitutive NF-κB activation Facilitates tissue damage	[162, 163]
GM-CSF/G-CSF	Promotes angiogenesis G-CSF contributes to myeloid derived suppressor cell recruitment at tumor site	[164, 165]
LCN2	Induces epithelial-mesenchymal transition (EMT) and promotes metastasis Promotes cell survival through iron sequesteration	[166, 167]
HBD1	Disrupts cell membrane and activates caspases in tumor cells Frequently lost in cancers, including prostate and renal cancers Recruits immature dendritic cell and memory T cell	[168, 169]

In malignant disease, increased expression of Th17 markers (IL-17A, RORα4 and RORγt) was observed in human lung AC compared to non-malignant lung tissue [103]. Th17 cells are key producers of IL-17, and this cytokine is known to contribute to the induction of lung cancer prometastatic factor expression. Elevated expression of IL-17 in peripheral blood was significantly correlated with TNM (tumor node metastasis) stage and increased expression of IL-17 receptor (IL-17RC) in NSCLC tumor cells was associated with invasive potential [104]. Treatment of NSCLC cell lines A549 and H520 with IL-17 resulted in increased phosphorylation of STAT3, which upregulated prometastatic factor production including vascular endothelial growth factor (VEGF) [105]. While IL-17 upregulation of STAT3 was shown to be mediated by IL-6 other cancer types [106, 107], this phenomena did not hold true in this case [105]. As a mechanism of lymph node metastasis, IL-17 can promote lymphangiogenesis by upregulating the expression of the lymphangiogenic factor vascular endothelial growth factor-C (VEGF-C) in murine lung cancer cells [108, 109] (Fig. 4). In humans, increased density of IL-17 positive cells in NSCLC tumors correlated with lymphatic vessel density [110]. Furthermore, loss of IL-17 has been shown to reduce metastases. Studies have shown that when challenged with Lewis lung carcinoma cells, IL-17 knockout mice had reduced number of metastatic nodules in lungs and improved survival compared to wild-type mice [104, 111]. Taken together, these studies reveal potential mechanistic and functional effects of IL-17 in cancer progression and metastasis.

Th17 cells produce other cytokines in addition to IL-17, including IL-22, which is also associated with lung cancer. IL-22 contributes to pro-survival signaling, angiogenesis and metastasis, part of which may be associated with its activation of STAT3 signaling pathway in cancer cells [112] (Fig. 4). High levels of IL-22 have been detected both locally in primary tumors and malignant pleural effusions (MPEs) and systemically in serum of NSCLC patients [113].

Th17 cells have complex biological functions and evidence suggests that these cells may paradoxically also contribute to anti-tumor immunity. With infusion of in vitro*-*generated Th17 cells, lungs of mice bearing B16 melanoma tumors had elevated dendritic cell and activated T cell recruitment, as well as elevated CCL20 and CCL2 expression, chemokines known to recruit these anti-tumor immune cells [114]. Increased IL-21 levels, also produced by Th17 cells, can induce tumor regression through expansion of CD8^+^ tumor-infiltrating lymphocytes in NSCLC as well as ovarian cancer and melanoma [115, 116] (Fig. 4). Moreover, high counts of pleural Th17 cells are associated with increased survival of NSCLC (lung AC and SqCC histologies) in human MPEs [117]. These studies reveal differential functions of Th17 cells, thus further investigation into their biological roles and clinical relevance in cancer development is warranted.

### Quantitative relationships between Th17s and Tregs in lung cancer prognosis

While various immune cell populations have been studied extensively in the context of cancer biology, the field has primarily focused on individual cell populations. More recently, cancer immunology has shifted towards a more integrated understanding of potential interactions among immune cell populations within the tumor microenvironment, including a focus on cellular ratios, crosstalk and phenotype plasticity in the context of cancer prognosis. Advancements in this framework include the development of an immunoscore as a potential component of cancer classification with prognostic relevance across a number of different tumor types, including NSCLC [118–121]. In addition to lung cancer, changes to Treg/Th17 levels have been observed in hematological and other solid tumor types in addition to autoimmune diseases and viral and bacterial infections, indicating that the balance of these two subsets plays an important role in regulating inflammation and cancer development [122–124]. Although both Treg and Th17 cells exert a diverse set of cancer-related functions (Figs. 3 and 4), these CD4^+^ T cell subsets may have opposing prognostic values in lung cancer, with a higher ratio of Tregs to Th17s correlating with more aggressive and advanced-staged malignancies [125, 126]. The balance of Treg and Th17 cells has been assessed in inflammation resulting from autoimmune disease, viral infections and bacterial infections [127–134]. In addition, Th17 and Treg cells are broadly considered to play pro- and anti-inflammatory roles, respectively, though it should be noted T cell plasticity allows for a functional continuum between these two CD4^+^ T cell subsets [135, 136]. In summation, the balance of these two CD4^+^ T cell subsets at local tumor and systemic sites appears to be strongly associated with lung cancer development, progression and prognosis.

The Treg/Th17 ratio and lung cancer prognosis has been assessed in peripheral blood. Peripheral blood of NSCLC patients is characterized by a significantly higher percentage of Th17 (CD4^+^IL-17^+^) and Treg cells (CD4^+^CD25^+^FoxP3^+^) compared to individuals without cancer. However, in NSCLC patients, the levels of these two CD4^+^ T cell subsets were inversely correlated in peripheral blood [137] (Fig. 6). Serum Th17 cells are known to positively correlate with IL-1β, IL-6, IL-23, while Tregs are known to positively correlate with TGF-β1 and IL-10 [137] (Fig. 6). NSCLC patients with Stage IV disease had higher Treg/Th17 ratios compared to patients with Stage I-III disease (Fig. 6). Conversely, the ratio was determined in another study to inversely correlate with serum levels of carcinoembryonic antigen (CEA), an oncofetal marker elevated in lung cancers with poor prognosis [137]. These studies highlight the variability of CD4^+^ T cell ratios in cancer progression, which certainly warrants further exploration [126].

In addition to assessing Treg/Th17 ratios in a systemic context in peripheral blood, proportions of these CD4^+^ T cell subsets have been characterized in biological fluids that are local to the lung, including pleural fluid (Table 2). Malignant and non-malignant pleural effusions (NMPE) derived from chronic inflammatory lung diseases, such as lung cancer and tuberculosis, have lower Treg and higher Th17 levels compared to non-chronic pleural effusions [138]. However, upon stimulation, CD4^+^ T cells in MPE secrete higher levels of IFN-γ, IL-6 and IL-17A and lower levels of IL-10 compared to that of non-malignant tuberculous pleural effusions (TPE) over time, suggesting that Th17s may maintain a pro-inflammatory environment in the pleural cavity of lung cancer patients [138] (Fig. 6). Conversely, an elevated Treg/Th17 ratio was found in MPEs when compared to NMPEs, such as parapneumonic pleural effusions (PPE) [139]. Furthermore, MPEs from lung cancer patients with a high Treg/Th17 ratio were found to correlate with poor survival [139]. These differences may be partially accounted for by the complex roles of inflammation in pro-tumor and anti-tumor activities during the course of cancer development [140].

**Table 2:** Characterization of Treg/Th17 ratios in malignant pleural effusions

Pleural effusion type compared	Observed characteristics of malignant pleural effusion (Relative to pleural effusion type compared)	References
Parapneumonic	**↑** Treg (CD4^+^CD25^+^FoxP3^+^)/Th17(CD4^+^IL-17^+^) ratio **↑** Foxp3/RORγt ratio	[139]
Malignant (with low Treg/Th17 ratio)	**↓** Overall survival with high Treg/Th17 in malignant pleural effusions	
Non-chronic diseases	**↓**Treg (CD4^+^CD25^+^CD127^low/−^)/Th17 (CD3^+^CD4^+^RORγt^+^) ratio ^a^ **↑** IL-17A^+^CD4^+^ cells **↓** CCR6^+^ Th17 cells	[138]
Tuberculous	No significant difference in Treg (CD4^+^CD25^+^CD127^low/−^)/Th17 (CD3^+^CD4^+^RORγt^+^) ratio **↑** IL-17A^+^CD4^+^ cells **↓** CCR6^+^ Th17 cells	

^a^ For consistency, cell ratios are presented as Treg/Th17

As evidenced by the studies mentioned above, the Treg/Th17 ratio has been primarily assessed in peripheral blood and pleural effusion samples. Collection of liquid biopsies requires less invasive procedures and are advantageous for longitudinal studies, as sampling can be conducted at multiple time points [141]. Future longitudinal studies will be very valuable to elucidating potential temporal relationships of these cell types during lung cancer pathogenesis and their clinical relevance. Treg and Th17 cell levels have been assessed in lung tumor biopsies, although these cell types are typically assessed individually using single markers on tissues sections, and Treg/Th17 ratios have not yet been reported for lung tumors. Part of the difficulty in assessing Tregs and Th17 cells in solid tumors is that both cell types are most accurately identified using multiple phenotypic markers and are therefore more amenable to analyses by single cell analytical methods, such as flow cytometry [142]. The advancement of multispectral imaging analyses of tissue sections may allow for Treg/Th17 ratio assessment in lung tumour biopsies in the future, while also providing extended insights into the clinical relevance of the spatial relationships of immune cells within the tumor microenvironment [143, 144].

While Th17s and Tregs have unique roles in the tumor microenvironment, interpretations of this ratio may be confounded by the presence of IL-17^+^FoxP3^+^ T cells, an intermediate Treg/Th17 phenotype that may be relevant to tumorigenesis [145] (Fig. 6). IL-17^+^FoxP3^+^ T cells may be generated from cytokine-dependent reprogramming of Tregs [146]. IL-17^+^FoxP3^+^ T cells have been implicated in autoimmune disease and solid cancer development, including inflammatory bowel disease and esophageal, colon and lung cancers [147–150]. In lung cancer, a CD45RA^−^CD45RO^+^FoxP3^hi^ subset enriched in NSCLC is characterized by increased RORγt and IL-17 expression [151]. Elucidation of specific molecular features and functions that IL-17^+^FoxP3^+^ T cells possess may reveal how this intermediate cell type influences inflammation in the tumor microenvironment, and may improve understanding of the Treg/Th17 ratio in cancer prognosis. The link between the balance of Tregs/Th17 cells and lung cancer prognosis underscores the clinical relevance of these cell types as biomarkers and potential therapeutic targets. With the current development of Th17 and Treg-targeted therapies, further studies assessing the complex roles of these immune cell types in lung cancer are needed if they are to be implemented in the clinic as a novel lung cancer treatment strategy [152–154].

## Conclusions

Inflammation mediated by infiltrating immune cells plays a key role in cancer pathogenesis. Among these, T cells display critical and diverse roles in the establishment and suppression of inflammation within the tumor microenvironment. These include Tregs and Th17s, CD4^+^ T cells which have been observed to mechanistically promote tumorigenesis, cancer progression and metastasis through immunosuppressive and pro-inflammatory functions. Specifically, Treg and Th17 cells in the tumor microenvironment modulate cytokine and chemokine production, promote immune cell recruitment and help regulate anti-tumor and pro-tumor immune cell activation states. Altered levels of these immune cell populations and their respective functions can facilitate lung cancer progression and metastasis. Furthermore, the balance of these CD4^+^ T cell populations at local and systemic sites may be clinically relevant in evaluating lung cancer prognosis. Further investigations are warranted to fully characterize the mechanistic effects and prognostic value of these immune cell populations in the context of cancer.

## Abbreviations

AC: Adenocarcinoma
BAL: Bronchoalveolar lavage
CCL: Chemokine (C-C motif) ligand
CEA: Carcinoembryonic antigen
COPD: Chronic obstructive pulmonary disease
COX2: Cyclooxygenase 2
CRP: C-reactive protein
CSF: Colony-stimulating factor
CTL: Cytotoxic T lymphocyte
CXCL: Chemokine (C-X-C motif) ligand
IFN-γ: Interferon gamma
IL: Interleukin
JAK/STAT: Janus kinase/signal transducer and activator of transcription
MPE: Malignant pleural effusion
NF-κB: Nuclear factor kappa-light-chain-enhancer of activated B cells
NK: Natural killer
NNK: Nicotine-derived nitrosamine ketone
NSCLC: Non-small cell lung cancer
NTHi: Nontypeable *Haemophilus influenzae*
RORγt: Retinoic acid receptor-related orphan receptor gamma t
ROS: Reactive oxygen species
SCLC: Small cell lung cancer
SqCC: Squamous cell carcinoma
TGF-β: Transforming growth factor beta
Th: T helper
